# ZBED Evolution: Repeated Utilization of DNA Transposons as Regulators of Diverse Host Functions

**DOI:** 10.1371/journal.pone.0059940

**Published:** 2013-03-22

**Authors:** Alexander Hayward, Awaisa Ghazal, Göran Andersson, Leif Andersson, Patric Jern

**Affiliations:** 1 Science for Life Laboratory, Department of Medical Biochemistry and Microbiology, Uppsala University, Uppsala, Sweden; 2 Science for Life Laboratory, Department of Animal Breeding and Genetics, Swedish University of Agricultural Sciences, Uppsala, Sweden; University of Lausanne, Switzerland

## Abstract

*ZBED* genes originate from domesticated *hAT* DNA transposons and encode regulatory proteins of diverse function in vertebrates. Here we reveal the evolutionary relationship between *ZBED* genes and demonstrate that they are derived from at least two independent domestication events in jawed vertebrate ancestors. We show that ZBEDs form two monophyletic clades, one of which has expanded through several independent duplications in host lineages. Subsequent diversification of *ZBED* genes has facilitated regulation of multiple diverse fundamental functions. In contrast to known examples of transposable element exaptation, our results demonstrate a novel unprecedented capacity for the repeated utilization of a family of transposable element-derived protein domains sequestered as regulators during the evolution of diverse host gene functions in vertebrates. Specifically, ZBEDs have contributed to vertebrate regulatory innovation through the donation of modular DNA and protein interacting domains. We identify that C7ORF29, ZBED2, 3, 4, and ZBEDX form a monophyletic group together with ZBED6, that is distinct from ZBED1 genes. Furthermore, we show that ZBED5 is related to Buster DNA transposons and is phylogenetically separate from other ZBEDs. Our results offer new insights into the evolution of regulatory pathways, and suggest that DNA transposons have contributed to regulatory complexity during genome evolution in vertebrates.

## Introduction

Transcriptional regulation is of critical importance to the development of genome complexity in multicellular organisms with differentiated cell types. Modulation of transcription is fundamental for facilitating spatial and temporal cellular specialization, and promoting phenotypic complexity. Understanding the evolution of regulatory networks is therefore a key research priority in genome biology [1].

**Figure 1:**
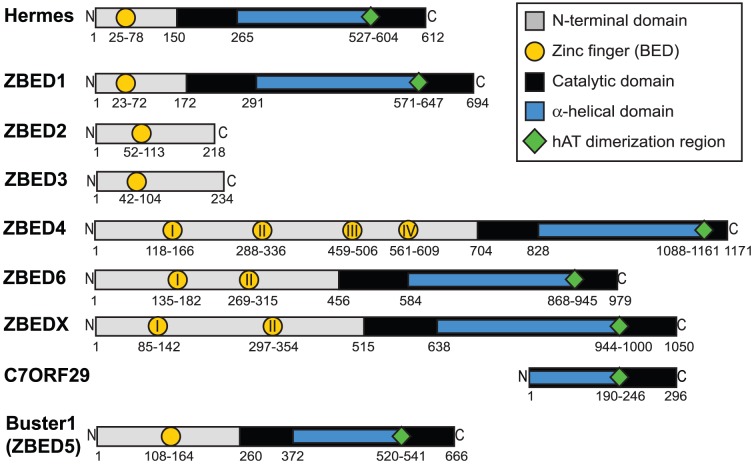
Schematic representation of ZBED, ZBEDX and C7ORF29 proteins. Coloured symbols correspond to different protein domains. Numbers represent estimated domain boundaries in amino acid positions. Roman numerals are used to indicate multiple BED domains within ZBED proteins. The housefly (*Musca domestica*) Hermes transposon serves as a reference for the schematics of human (*Homo sapiens*) ZBED1-4 and 6, western clawed frog (*Xenopus tropicalis*) ZBEDX, and human Buster1 (ZBED5). C7ORF29 is present in mammals and is syntenic with ZBEDX but lacks the N-terminal domain and part of the catalytic and alpha-helical domains (human and chimpanzee (*Pan troglodytes*) are also further truncated at the dimerization domain for C7ORF29).

A central mechanism of transcriptional regulation occurs via the action of DNA-binding transcription factors through their interaction with regulatory DNA sequence motifs. These transcription factors may activate or repress transcription, and can influence single phenotypic traits or control entire pathways. The *ZBED* gene family is a group of closely related genes that encode proteins involved in the regulation of diverse functions in vertebrates. A recently identified example of a transcription factor from this family that regulates diverse phenotypic effects is ZBED6 in placental mammals. ZBED6 binds a conserved target motif and thereby represses the expression of insulin-like growth factor 2 (*IGF2*). A single nucleotide substitution in intron 3 of *IGF2* in pigs leads to a disruption of ZBED6 binding affecting development, cell proliferation, wound healing, and muscle growth [2], [3]. Based on sequence similarity and protein domain architecture (Fig. 1), it was postulated that ZBED6 is derived from a *hAT* superfamily DNA transposable element [4], suggesting exaptation by the host genome [2].

Transposable elements (TEs), such as DNA transposons and retrotransposons, are major components of many eukaryotic genomes and typically constitute large proportions of vertebrate genomes [5]–[7]. It has been shown that numerous genes contain functionally important TEs (particularly those with rapidly evolving coding sequences), which alter gene regulation and expression [6], [8], [9]. For example, TE-derived sequences are found in around one quarter of analyzed human promoter regions and appear to function as alternative promoters for many genes [8]. In the human genome around 7 Mb sequence representing some 280,000 regulatory elements have been reported to originate from insertions of mobile DNA [10]. As with ZBED6, TEs can also contribute entire functional genes to the host genome through an evolutionary process known as ‘molecular domestication’ [6], [11], [12]. Domesticated TEs are no longer mobile and are often present as single-copy orthologues in the genomes of related organisms [11].

Here, we use phylogenetic analyses to explore the relationship of *ZBED* genes to DNA transposable elements of the *hAT* superfamily [4], with which they show high sequence and structural similarity (Fig. 1 and REF [13]). In a previous study, Aravind performed sequence analyses and described a protein signature, Cx_2_Cx_n_Hx_3–5_[H/C] predicted to form a zinc finger, shared among plant, animal and fungal proteins [13]. The protein domain, named the BED finger after the domesticated *Drosophila* BEAF and DREF proteins, was predicted to either have been acquired by transposons from cellular genes or more probably recruited for cellular functions from transposases on one or two independent occasions [13]. Using phylogenetic methodology, we have tested these predictions and investigated the evolutionary history of all currently identified *ZBED* genes, containing varying numbers of BED domains.

*ZBED* genes are widely expressed among vertebrate tissues and together they regulate a remarkable diversity of functions. ZBED1 regulates transcription of multiple ribosomal protein genes and is linked to cell proliferation [14]. ZBED3 is an axin-interacting protein important for *Wnt*/*β-catenin* signal modulation, involved in embryogenesis and carcinogenesis in mammals [15]. ZBED4 contains a nuclear hormone receptor interacting motif, and is localized to cone photoreceptors and glial Müller cells in the retina. It is also predicted to interact with hormone pathways in the ovary and several other tissues [16]. ZBED6 acts as a repressor at the *IGF2* locus and ChIP-seq data indicate that it has many other target sites in the genome of placental mammals [2]. The functions of ZBED2, ZBEDX and C7ORF29 (a novel ZBED family member identified here) remain to be elucidated.

Currently, it is unclear how closely related ZBEDs are to one another relative to other sequences in the *hAT* transposon superfamily, and whether separate domestication events have contributed to *ZBED* gene diversity versus gene duplications within the host lineage. The *hAT* transposons and related domesticated sequences constitute a large superfamily that was recently characterized and divided into the *Ac* and *Buster* families, named after the first identified transposon or transposon-like sequence in each family, with support from differences in target-site selections generated by active transposons and phylogenetic analysis [4]. Through comparisons with active DNA transposons and related sequences from the *Ac* and *Buster* families of the *hAT* transposon superfamily, we clarify the history of ZBED domestication and reveal the evolutionary relationships of *ZBED* genes.

## Results and Discussion

**Figure 2:**
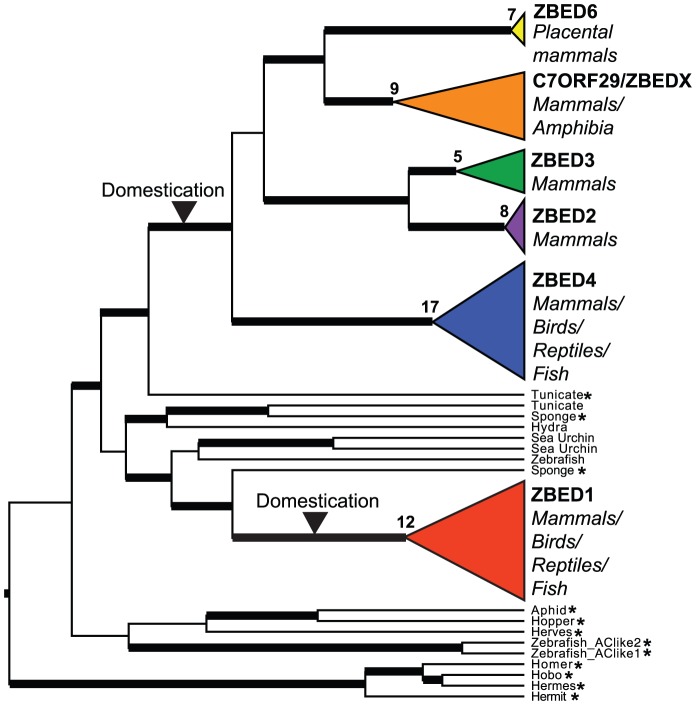
ZBED evolution. Phylogenetic tree for *ZBED* genes and related sequences from the *Ac* family. Two separate ZBED domestications are indicated. Numbers of included taxa are provided next to schematic clades. Active DNA transposons are marked with asterisks, and bold branches indicate posterior probabilities ≥95%.

**Figure 3:**
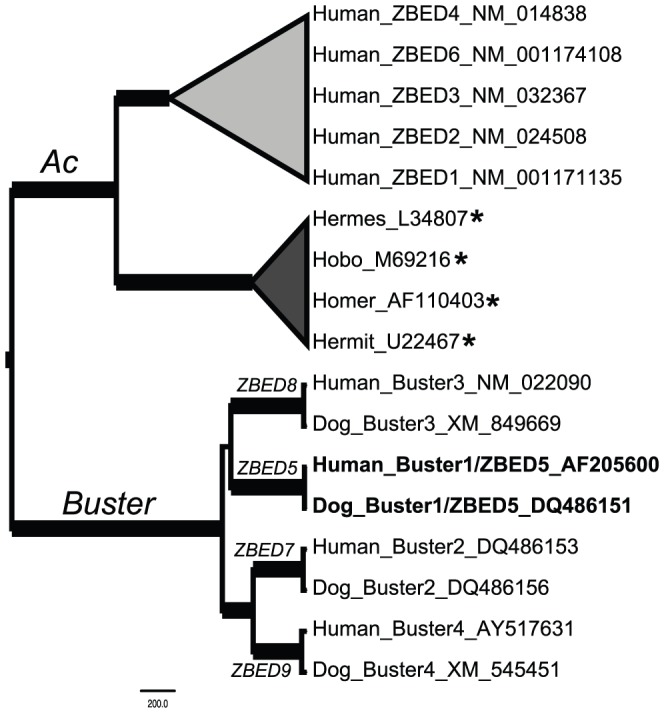
Phylogenetic relationships of Buster1 (ZBED5). ZBED5 is identical to Buster1 and groups within the *Buster* family with strong support. Buster sequences are separate from collapsed clades representing the *Ac* family. Active DNA transposons are marked with asterisks, and bold branches indicate posterior probabilities ≥95%. Proposed nomenclature updates for ZBEDs 7, 8 and 9 are indicated next to branches ancestral to the respective ZBED (Buster) clade.

In accordance with previous predictions that BED domains may have been domesticated from transposases on one or two independent occasions [13], our phylogenetic analyses demonstrate that ZBEDs form two monophyletic clades within the *Ac* transposon family (Fig. 2), except ZBED5, which instead belongs within the related *Buster* family and is separate from other ZBEDs (Fig. 3). *ZBED1* genes from multiple species form one clade. Additionally, a close evolutionary relationship between C7ORF29 and ZBED6, ZBED2, and ZBED3 is identified. Table 1 shows the chromosomal location of each *ZBED* gene with reference to the human genome, details of the integration landscape and confirmed orthologous *ZBED* synteny in other species.

**Table 1:** Chromosomal locations of human *ZBED* genes, frog *ZBEDX* and human Buster1 (*ZBED5*).

Name	Chromosomal location^a^	Integration landscape	Orthologues	Confirmed Synteny
ZBED1^b^	Chr. X: 2,404,529–2,419,049	DHRSX (intron 1)	Cow, Horse, Panda, Dog, Platypus, Chicken, Zebrafinch, Frog, Lizard, Fugu, Stickleback	All orthologues are located at variable distances upstream of DHRSX
ZBED2	Chr. 3: 111,311,747–111,314,182	CD96 (intron 5)	Cow, Horse, Pig, Panda, Dog, Sloth^e^, Armadillo^e^	All orthologues
ZBED3	Chr. 5: 76,372,532–76,383,030	Intergenic (downstream of AGGF1)	Cow, Pig, Mouse, Sloth^e^	All orthologues
ZBED4	Chr. 22: 50,247,497–50,283,726	Intergenic (upstream of ALG12)	Cow, Horse, Panda, Dog, Dolphin^e^, Mouse, Pig, Chicken, Zebrafinch, Frog, Lizard, Fugu, Stickleback, Medaka, Tilapia^e^, Zebrafish	All orthologues are located in an intergenic region either upstream or downstream of ALG12
ZBED6	Chr. 1: 203,766,651–203,769,590	ZC3H11A (intron 1)	Cow, Pig, Horse, Panda, Dog, Mouse	All orthologues
C7ORF29	Chr. 7: 150,026,938–150,029,811	LRRC6 (intron 2)	Pig, Horse, Panda, Dog, Chimp, Platypus, Frog	Chimp is also located in intron 2, all other orthologues are located upstream of LRRC61
ZBEDX^c^	GL173523: 210,296–213,445	Intergenic (upstream of LRRC61)	N/A	N/A
Buster1 (ZBED5)^d^	Chr. 11: 10,874,251–10,879,620	Intergenic (downstream of EIF4G2)	Dog	Orthologue is syntenic

a Positions in the human genome (hg19 assembly), and the frog genome (*Xenopus tropicalis*, xenTro3 assembly).

b Also referred to in the literature as hDREF, Tramp, and Human-*Ac*.

c The frog *ZBEDX* gene.

d This gene is also referred to as Buster1 of the *Buster* DNA transposon family in the literature, and is distinct from other ZBEDs.

e Sequence conversion is not currently available for these genomes in the UCSC Genome Browser (http://genome.ucsc.edu).

Active *Ac* TEs from plant and invertebrate genomes occur ancestrally to the ZBEDs, while sequences from diverse invertebrate taxa and zebrafish fall between the two monophyletic ZBED clades (Fig. 2). This suggests *ZBED* genes originate from at least two independent *hAT* DNA transposon domestication events in a primitive jawed-vertebrate ancestor, since no ZBEDs were identified in jawless fish (lamprey and hagfish), or in more primitive vertebrates. The pattern is also consistent with molecular clock estimates for coalescent dates among ZBEDs (Fig. S1). The structural variation observed among ZBEDs (Fig. 1) indicates successive usage of DNA TE-derived protein domains for regulatory purposes within host genomes via duplication (Fig. 2) followed by functional diversification. Bayesian Inference (BI) and Maximum Likelihood (ML) DNA and amino acid trees show highly similar topologies, a difference being that ZBED2 sequences do not form a monophyletic clade in ML analyses, instead placing more basally to the ZBED3 clade (BI DNA in Fig. 2, ML DNA in Fig. S3, and ML amino acid in Fig. S4).

As indicated above, we find that the *Buster1/ZBED5* gene belongs to the Buster family of DNA TEs and related sequences, in contrast with other *ZBED* genes that belong to the *Ac* family (Fig. 3). Since the BED domain is shared among sequences in both the *Ac* and *Buster* families (Fig. 1), we suggest that domesticated elements from the *Ac* and *Buster* families are collectively referred to as *ZBED* genes. Thus, *ZBED* genes in the *Ac* family will retain their current nomenclature, while *Buster2-4* will be relabeled as *ZBED7-9*. This naming system separates *Ac* and *Buster* derived genes that were domesticated in ancestral vertebrate lineages from active *hAT* superfamily transposons and those domesticated in isolated invertebrate taxa (see Fig. 2 and Fig. 3). The suggested modification to nomenclature minimizes the potential for confusion considering that members of the *hAT* superfamily are already rich in synonyms. For example, ZBED1 is also known as TRAMP, human-*Ac*, and hDREF, despite a lack of close phylogenetic relationship to either the DREF or *Ac* elements (REF [4] and this study).

The *C7ORF29* locus, which is present in multiple mammalian genomes (Fig. 2), shares sequence similarity with the 3′ region of the newly identified *ZBEDX* gene, currently only known from *Xenopus tropicalis*. However, while the predicted C7ORF29 molecule is truncated to a short segment of the catalytic domain (Fig. 1), *ZBEDX* coding sequence extends ∼2 kb in the 5′ direction and contains an N-terminal domain with two BED domains. Despite the apparent truncation of C7ORF29 and loss of its BED domains, sequence conservation and the presence of an open reading frame in all taxa included here argues that it may be expressed and under functional constraint. The *Xenopus ZBEDX* gene was identified using sequence similarity searches in ENSEMBL and NCBI using the *ZBED6* gene as a query. *ZBEDX* is not orthologous to other *ZBED* genes but is syntenic with the truncated *C7ORF29* locus in the human genome.

In recent years the importance of transposase domestication for host genome functions has become evident. An example being the *RAG1/2* genes, suggested to be domesticated *Transib* DNA TEs, that catalyze DNA cleavage during V(D)J recombination of *Immunoglobulin* genes for specific immune responses to foreign antigens [17]. In addition to our study, other examples of protein domain families hypothesized to be derived from transposon domestication events [18], [19], as well as other examples of multiple independent domestications of related transposons have been reported [20], [21]. However, identification of a family of TE-derived genes that regulate multiple diverse and fundamental functions, as described here for ZBEDs in vertebrates, is unusual and provides further support for the importance of TEs in the evolution of host genome function.

Definition of the factors that predispose certain TE-derived sequences to become significant players in host genome regulation is of crucial importance. Transposases may be uniquely suited for exaptation to host regulatory functions due to potential selective co-domestication of TE binding site networks derived from related elements throughout the genome [22]. Transposases also contain DNA binding and catalytic domains that enable shuffling and duplication of DNA which may present opportunities for structural modifications of the genome. The tree topology (Fig. 2) and *ZBED* gene distribution among taxa imply successive *ZBED* duplications during vertebrate evolution, with domain gains/losses following duplications, suggesting an inherent suitability of ZBED domains in host genome functions. The observed ability of closely related ZBED molecules to regulate highly divergent host functions is intriguing, and suggests a particular suitability of ZBED protein domains for host functions. Specifically, ZBEDs contain a zinc finger DNA binding domain, distinct from the classical BED domain (Fig. 1) [13]. They also share a conserved DDE amino acid motif within a region corresponding to the catalytic domain, similar to that observed in integrases of retroviruses and LTR-retrotransposons [23].

**Figure 4:**
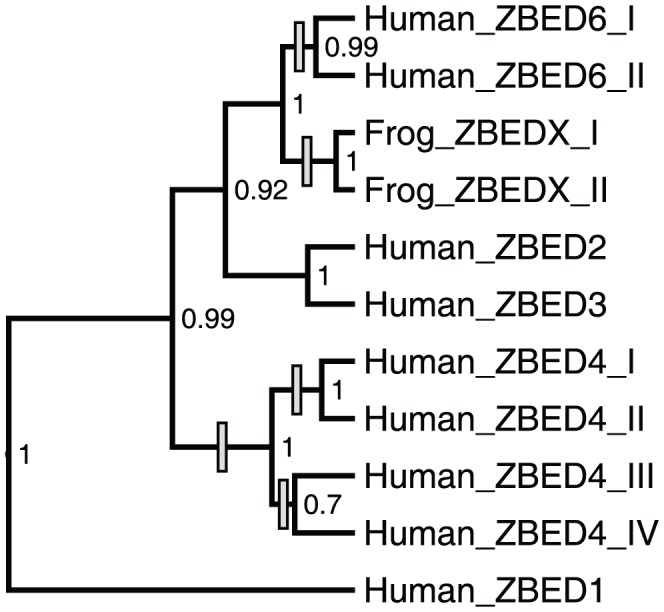
Phylogenetic relationships of separate BED domains. Roman numerals refer to BED domain position within ZBED genes (Fig. 1). Grey boxes on branches indicate hypothesized BED domain duplication events for the various *ZBED* genes. Posterior probabilities are provided next to tree nodes.

The number of BED domains varies among ZBED proteins, with ancestral active transposons and ZBED1 containing a single BED domain, while ZBED6 and ZBEDX contain 2 BED domains, ZBED4 contains 4 BED domains, and C7ORF29 has lost the BED domain (Fig. 1 and Fig. 2). To investigate evolutionary events leading to this variation in numbers of BED domains among the ZBEDs, we analyzed an alignment of ZBED-derived BED domains to test for possible recombination and duplications (Fig. 4). Our results suggest multiple independent duplications of sequences encoding BED domains after *ZBED* gene domestication (excluding full *ZBED* gene duplication events, see Fig. 2), with no evidence of recombination (no occurrences of close similarity between BED domains from separate *ZBED* genes, see Fig. 4). Our analyses demonstrate that this important domain originated from a TE sequence, and was subsequently exapted for diverse and fundamental host functions in vertebrate lineages. Further functional and structural analyses of ZBED proteins may help to identify the advantages offered by these protein domains, allowing insights into why they have been repeatedly utilized for host regulatory purposes.

A recently domesticated DNA transposon is expected to be selectively neutral and show mutational drift within the host genome, unless harmful or beneficial for host function. DNA transposons may be suited to domestication for host functions, given that they typically encode multi-domain proteins with diverse functions including DNA and protein binding affinities. In such cases, the evolution of domesticated sequences is expected to reflect their new role. For example, the zinc finger containing poly-ZF family of putative transcriptional repressors, which have been hypothesized to defend against viruses and/or transposons, show signatures of adaptive evolution as expected for genes subject to ongoing positive selection [19]. In contrast, adaptive evolution may occur early in the domestication process and be followed by long periods of stabilizing selection, as observed for the mammalian centromere-associated protein-B [20]. Based on our phylogenetic results and given the proposed functions of ZBEDs we find no evidence that they are subject to ongoing positive selection. Analyses of selection along ZBED domestication branches (Fig. 2) versus all other branches did not show a significant difference in selection pressure (ω, dN/dS). Attempts to implement more powerful branch-site models produced misleading results (PAML model 2, branch-site selection test: ω = 999, no sites identified under Bayes Empirical Bayes estimation). These results are possibly due to high levels of sequence divergence and saturation of synonymous mutations, which do not preclude phylogenetic inference but are problematic for analyses of adaptive evolution using existing models. The majority of sites in our DNA alignment are divergent and just 2.6% are fixed across all lineages (Fig. S2). Our phylogenetic results are also complicated by long branch lengths, with molecular clock estimates suggesting ZBED domestication occurred several hundred million years ago (Fig. S1). Large portions of *ZBED* genes may have evolved under conditions of relaxed evolutionary constraint during their history, while certain structural properties and key motifs were maintained. Thus, underestimation of synonymous mutations may hinder the ability to discern sites that truly underwent positive selection. However, it is possible that information currently missing from the evolutionary record together with refined models may improve resolution in selection analyses.

It is intriguing that we observe conservation of the DDE amino acid triad (see above), which forms a catalytic pocket during DNA cleavage in active DNA transposons [24], [25] across domesticated *ZBED* genes. This suggests an unknown role for the DDE triad in ZBEDs other than a cut and paste function. It is notable that the previously described alpha helical domain inserted into the Hermes catalytic domain [24] is also present in domesticated ZBEDs (Fig. 1) and in the Buster family [4], suggesting that this structural modification involving spacing of the DDE triad was introduced early or before *hAT* transposon evolution (Fig. 2 and Fig. S1).

DNA TEs are found across Prokaryota and Eukaryota and most likely diversified early in the history of life. Evidence suggests they have acted as significant drivers of complexity during genome evolution. Currently, we have a limited understanding of how active and domesticated TEs interact with each other and with the host's genome at the molecular level. Furthermore, what factors predispose certain transposons to domestication, such as elements from the *Ac* and *Buster* families, and the mode by which they are domesticated as regulators in host genomes are largely unknown. Further studies examining the nature of the molecular interactions between these transposon-derived protein domains and target DNA sequences are required. Developments in this field are likely to offer considerable scope for novel applications arising from functional effects exerted by DNA transposons, and a deeper understanding of the evolution of complex gene regulatory networks. As a consequence, our findings have implications for current understanding of the origin and operation of complex genomic regulatory functions, and knowledge of the functionality and efficacy of DNA-protein interacting domains.

## Methods

Sequences were retrieved using known ZBED sequences in BLAST searches at NCBI (http://blast.ncbi.nlm.nih.gov/Blast.cgi) against nucleotide, EST, and reference genome databases. Additional sequences were retrieved from REPBASE (http://www.girinst.org/repbase/), TEFam (http://tefam.biochem.vt.edu) and ENSEMBL (http://www.ensembl.org). For *ZBED* genes, taxa were restricted to a sample representative of major vertebrate lineages. Syntenic genomic positions of *ZBEDs* were confirmed using the UCSC genome browser (http://genome.ucsc.edu/). Protein domains were identified using InterProScan (http://www.ebi.ac.uk/Tools/pfa/iprscan/) with reference to the crystal structure identified for the related Hermes *hAT* superfamily (*Ac* family) DNA transposon [24]. Putative active (or recently active) transposons were identified by searching 500 nucleotide (nt) flanking sequences for evidence of Target Site Duplications (TSDs) and Terminal Inverted Repeats (TIRs), using online tools (http://mobyle.pasteur.fr/cgi-bin/portal.py?#forms::palindrome) and manual confirmation.

Amino acid sequence alignments were constructed in ClustalX 2.1 [26] and MUSCLE 3.7 [27] and edited using Jalview 2.7 [28] and MEGA 5.0 [29]. In cases where multiple BED domains are present in the same *ZBED* gene, their sequences show high similarity and are more closely related to each other than to BED domains from other ZBED genes (see Fig. 4). Thus, given that the ancestral state appears to be a single BED domain (as for active *Ac* transposons, and ZBEDs 1, 2, 3, and 5), for consistency only the furthest downstream BED domain was retained for alignment purposes. A nucleotide alignment was generated from the amino acid alignment using a custom Perl script. This procedure was more robust than constructing a multiple alignment directly from DNA sequences, given the level of divergence observed for sequences included in this study (see Fig. S1). Two regions of low conservation, for which it was not possible to infer true homology, were excluded to produce a 1983 nt alignment for subsequent phylogenetic analyses. The first omitted 192 nt segment is located immediately downstream from the start of the identified catalytic domain and the second omitted 297 nt segment is located immediately upstream of the *hAT* dimerization region. Several phylogenetically unstable taxa with excessively long branch lengths were identified and removed from the alignment during initial analyses. The final DNA alignment is provided in the supporting information (Fig. S2).

Phylogenetic relationships were estimated using Bayesian Inference implemented in BEAST [30] and Maximum Likelihood implemented in RAxML [31]. Nucleotide analyses in BEAST were run using the SRD06 model [32], which specifies the HKY substitution model [33], with four gamma rate categories, and two codon partitions (codon positions (1+2), and 3). A strict molecular clock was implemented by specifying a normally distributed prior utilizing a frequently applied neutral rate estimate for mammalian coding sequence as the mean [34]. Analyses were initiated from random trees, and the final analysis ran for 10,000,000 generations. Nucleotide analyses in RAxML were run using the GTRGAMMA model with four gamma rate categories and two codon partitions (codon positions (1+2), and 3), and initiated from random starting trees. A rapid bootstrap analysis was conducted for the best-scoring ML tree with 1,000 replicates. RAxML amino acid analyses were run using the PROTCATJTT model with empirical base frequencies, and similar starting tree and bootstrap settings.

Analysis of selection among sequences was measured using a maximum likelihood approach implemented in the codeml program of PAML version 4.5 [35], [36]. Codeml branch model analyses were performed under one-ratio, free-ratio, and two-ratio models specifying domestication branches, and initiated multiple times using a range of starting values for κ and ω. In an attempt to resolve long phylogenetic branches (see Fig. S1) and saturation of synonymous mutations in our estimates of selective pressure, we implemented the more powerful Codeml branch-site model, in each case specifying branches ancestral to the following clades **1:** *(ZBED6)*, **2:** *(C7ORF29/ZBEDX)*, **3:** *(ZBED6/C7ORF29/ZBEDX)*, **4:** *(ZBED3)*, **5:** *(ZBED2)*, **6:** *(ZBED2/ZBED3)*, **7:** *(ZBED6/C7ORF29/ZBEDX/ZBED2/ZBED3)*, **8:** *(ZBED4)*, **9:** *(ZBED6/C7ORF29/ZBEDX/ZBED2/ZBED3/ZBED4)*, and **10:** *(ZBED1)* (compare to Fig. 2). Results were compared using likelihood ratio tests.

## Supporting Information

Figure S1 **ZBED evolution.** Bayesian strict clock phylogeny showing 95% Highest Posterior Density estimates for coalescent dates among *ZBED* genes. Posterior probabilities are indicated next to nodes. Estimated coalescence intervals are indicated within parentheses and the scale is in millions of years.
(PDF)

Figure S2 **DNA alignment of ZBED and related sequences analyzed in this study.**
(TXT)

Figure S3 **Maximum Likelihood DNA tree.**
(PDF)

Figure S4 **Maximum Likelihood amino acid tree.**
(PDF)
