# Supplementary figures and images for: ZBED Evolution: Repeated Utilization of DNA Transposons as Regulators of Diverse Host Functions
Source: PLoS One. 2013 Mar 22;8(3):e59940. doi: 10.1371/journal.pone.0059940 (PMC3606216; doi:10.1371/journal.pone.0059940)

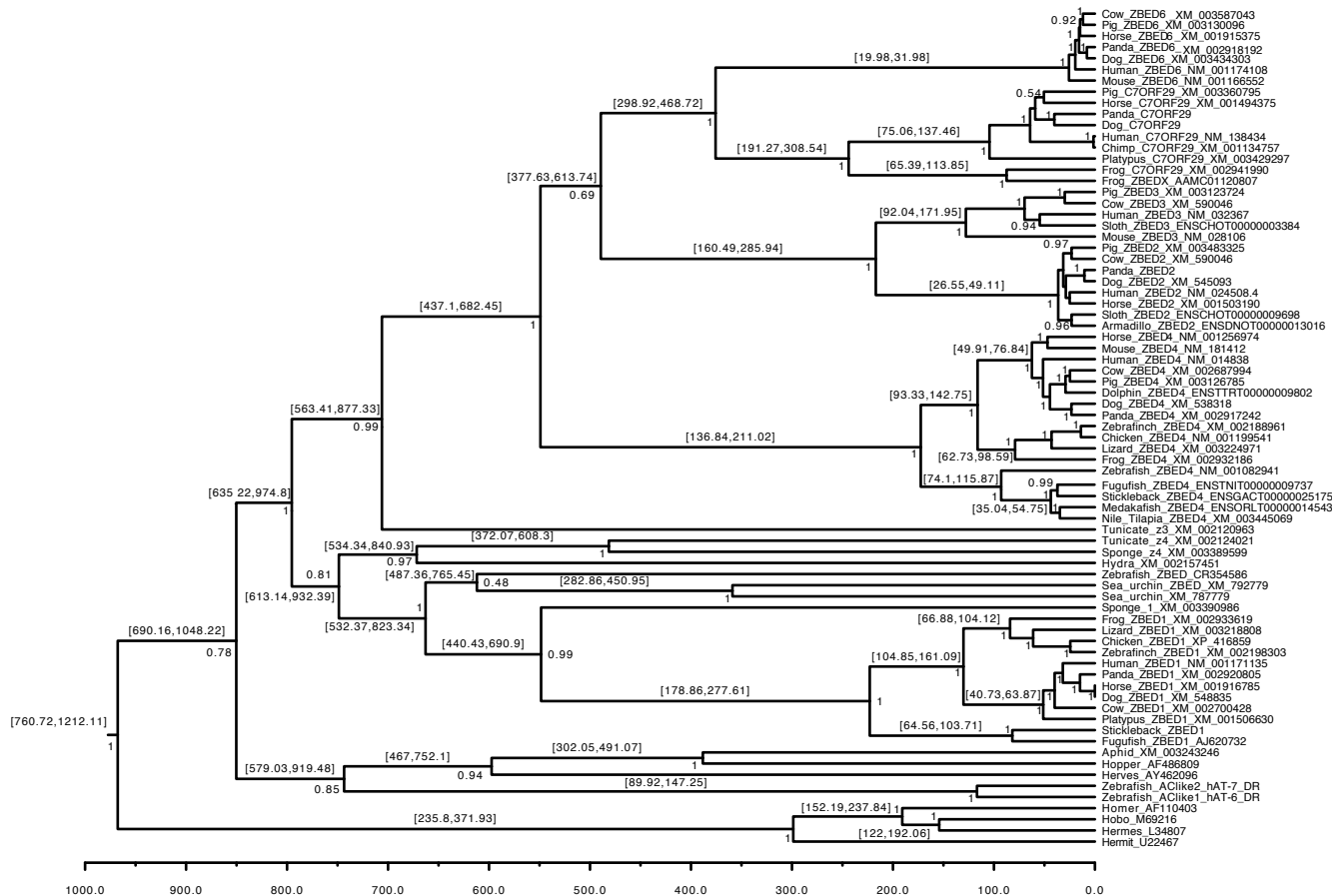

Supplement: Figure S1 — ZBED evolution. Bayesian strict clock phylogeny showing 95% Highest Posterior Density estimates for coalescent dates among ZBED genes. Posterior probabilities are indicated next to nodes. Estimated coalescence intervals are indicated within parentheses and the scale is in millions of years. (PDF) [file pone.0059940.s001.pdf]

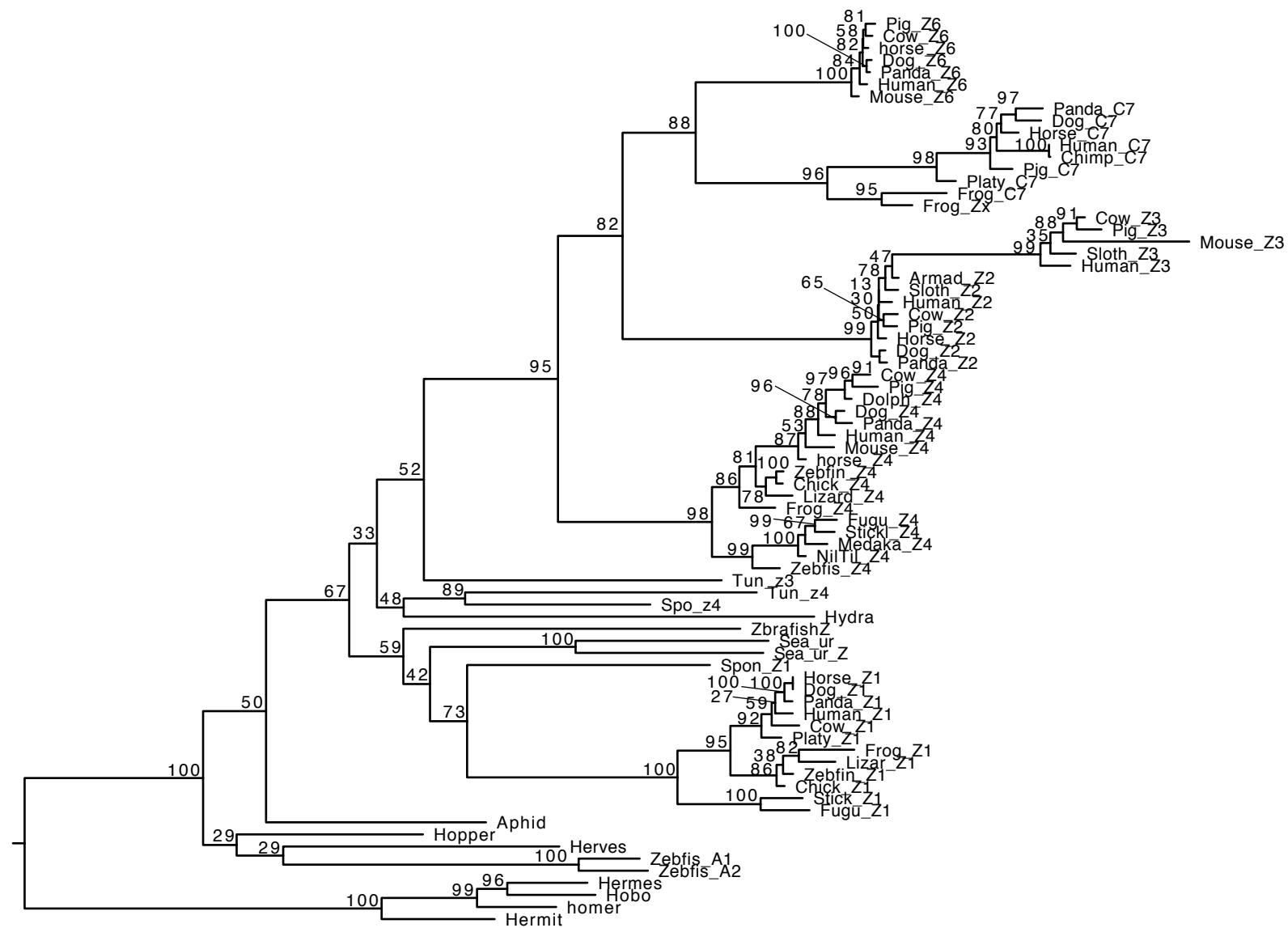

0.6

Supplement: Figure S3 — Maximum Likelihood DNA tree. (PDF) [file pone.0059940.s003.pdf]

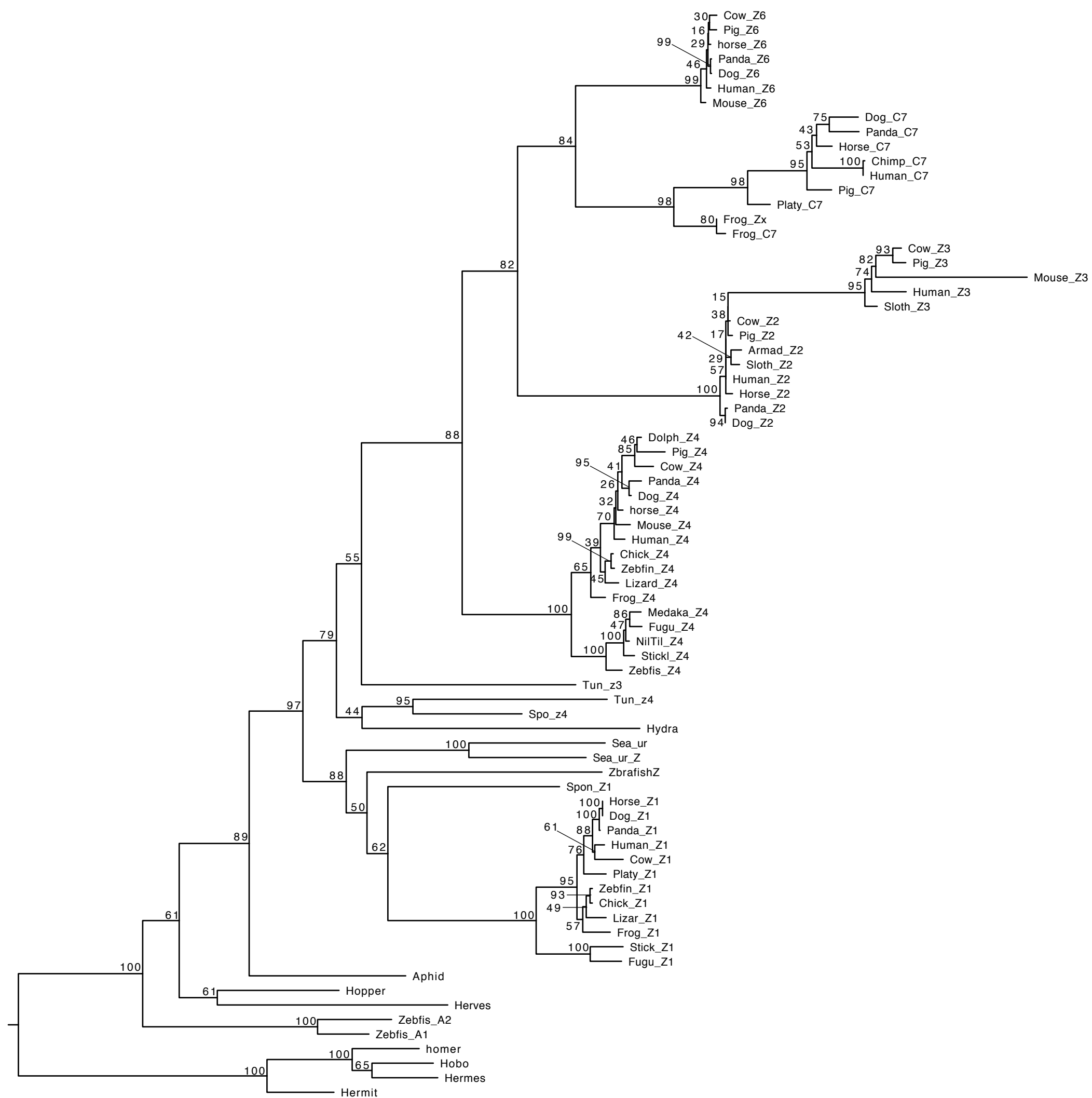

Supplement: Figure S4 — Maximum Likelihood amino acid tree. (PDF) [file pone.0059940.s004.pdf]
